# Critical Appraisal of Amyloid Lowering Agents in AD

**DOI:** 10.1007/s11910-021-01125-y

**Published:** 2021-06-10

**Authors:** Boris Decourt, Fadel Boumelhem, Evans D. Pope, Jiong Shi, Zoltan Mari, Marwan Noel Sabbagh

**Affiliations:** 1 Cleveland Clinic Lou Ruvo Center for Brain Health, 888 W. Bonneville Ave, Las Vegas, NV 89106 USA; 2 UNLV School of Medicine, Las Vegas, NV USA

**Keywords:** Alzheimer’s disease, Amyloid beta, Immunotherapy, Monoclonal antibody therapy

## Abstract

**Purpose of Review:**

According to the amyloid cascade hypothesis, removing amyloid beta (Aβ) should cure Alzheimer’s disease (AD). In the past three decades, many agents have been tested to try to lower Aβ production, prevent Aβ aggregation, and dissolve Aβ deposits. However, the paucity in definitive preventative or curative properties of these agents in clinical trials has resulted in more avant-garde approaches to therapeutic investigations. Immunotherapy has become an area of focus for research on disease-modifying therapies for neurodegenerative diseases. In this review, we highlight the current clinical development landscape of monoclonal antibody (mAb) therapies that target Aβ plaque formation and removal in AD.

**Recent Findings:**

Multiple potential disease-modifying therapeutics for AD are in active development. Targeting Aβ with mAbs has the potential to treat various stages of AD: prodromal, prodromal to mild, mild, and mild to moderate. Monoclonal antibodies discussed here include aducanumab, lecanemab, solanezumab, crenezumab, donanemab, and gantenerumab.

**Summary:**

The final decision by the FDA regarding the approval of aducanumab will offer valuable insight into the trajectory of drug development for mAbs in AD and other neurodegenerative diseases. Future directions for improving the treatment of AD will include more inquiry into the efficacy of mAbs as disease-modifying agents that specifically target Aβ peptides and/or multimers. In addition, a more robust trial design for AD immunotherapy agents should improve outcomes such that objective measures of clinical efficacy will eventually lead to higher chances of drug approval.

## Introduction

Alzheimer’s disease (AD) is the most prevalent type of dementia, affecting 5.8 million people ages 65 and older in the USA alone, and this number is estimated to grow to 13.8 million by mid-century [1]. In other words, one in ten people ages 65 and older (10%) have AD in the USA [1]. Following the onset of AD dementia, the median survival time ranges from 3.3 to 11.7 years. [2]. Because the number of deaths continues to increase, and because of the tremendous financial burden to society, the need for disease-modifying treatments for AD is dire.

The two neuropathological hallmarks of AD are amyloid β (Aβ) plaques and neurofibrillary tangles (NFT) [3, 4]. NFTs consist of filamentous inclusions or aggregates of aberrantly misfolded and hyperphosphorylated tau proteins that accumulate intra-neuronally [3]. Aβ peptides (molecular weight ~4 kDa) are the product of the successive cleavage of amyloid precursor protein (APP; molecular weight ~ 120 kDa) by β- and γ-secretases, which is referred to as the amyloidogenic pathway [5]. Alternatively, APP can be first cleaved by an α-secretase inside the amyloid peptide sequence (i.e., amino acid 16), then γ-secretase to generates shorter soluble extracellular fragments, termed p3, that are thought to be non-amyloidogenic [5].

During the course of AD, Aβ peptides that are 36–43 amino acids long (e.g., Aβ_40_, Aβ_42_, Aβ_43_) can assemble into insoluble beta-sheet fibrillar aggregates that deposit extracellularly in the brain parenchyma and cerebral vasculature [6, 7]. Over time, amyloid plaques may grow from a single Aβ fibril extracellular nucleation site [8]. Amyloid deposition is accompanied with the disruption of synaptic structure and function, and neuronal atrophy often starting in the hippocampus area then spreading to cortical regions, which ultimately induces cognitive impairment and dementia symptoms [9–11]. Cerebral vasculature deposits (also known as cerebral amyloid angiopathy; CAA) can cause hemorrhages, strokes, and inflammation [6, 7]. The mechanism of toxicity of amyloid peptides is widely debated. The mechanism can be viewed either as a gain-of-toxicity in the amyloid peptides and their soluble misfolded oligomeric antecedents [12–14], or as a loss of function due to the adoption of misfolded conformations [15]. Some argue that Aβ is intrinsically toxic. Others suggest Aβ seeds the formation of tau tangles [14, 16].

According to the amyloid cascade hypothesis, which was introduced in 1992, deposition of Aβ peptides is the main causative agent of AD pathology, i.e., NFT, cell loss, CAA, vascular damage, and dementia follows as a direct result of this deposition [17]. Phenotypic heterogeneity of AD pathology is suggested to be induced by polymorphic Aβ fibrils that precipitate as heterogeneous plaque pathology, such as cored (mature) plaques and diffuse plaques [18–20]. Importantly, based upon the amyloid cascade hypothesis, the removal of brain Aβ plaques should stop the progression of AD. This concept stimulated the development and testing of highly innovative anti-Aβ therapeutic agents in the past three decades to lower Aβ production, prevent Aβ aggregation, and dissolve Aβ deposits. However, this idea is currently debated due to the failure of all clinical trials testing this concept until now [32657175].

Diffuse plaques are frequently observed in cognitively unaffected, amyloid positive (CU-AP) individuals [21]. Diffuse plaques have been found to have a capacity to convert into cored plaques in sporadic AD (s-AD) [21]. Maturation of diffuse into cored plaques in s-AD was found to correlate with increased deposition of Aβ_40_ at the center of the cored plaques [21]. Diffuse plaques in both s-AD and CU-AP are characterized by deposition of Aβ_42_ [21]. Moreover, diffuse plaques in s-AD show increased levels of pyroglutamate*-*modified N-terminally truncated Aβ_42_ species (N-pyro-E-Aβ; AβpE3-42, AβpE11-42) in comparison with diffuse plaques in CU-AP [21]. The correlation of s-AD with deposition of Aβ_40_ and the finding of increased diffuse Aβ_42-_rich plaques in CU-AP patients is contrary to the current understanding that Aβ_42_ is the major Aβ toxic species associated with AD pathogenesis, and suggests a more complex mechanism involving Aβ_40_ as well [21–24].

Aβ peptides vary in toxicity, with Aβ_43_ being the most cytotoxic and Aβ_40_ being the most benign as it is produced physiologically throughout life [5, 25–27]. Aβ_42_ demonstrates neurotoxicity and has the faster aggregation rate [28]. The most synaptotoxic species of Aβ are the small oligomers (2–10 monomers), and pyroglutamate forms of Aβ oligomers [29]. The current hypothesis is that both soluble and insoluble Aβ fibrils may contribute to the pathogenesis and progression of AD [28]. Thus, using Aβ lowering agents is still considered a viable approach as a disease-modifying treatment for AD. Below, we review the main Aβ lowering approaches tested in humans and an accurate update on their clinical testing.

### Aβ Synthesis Inhibitors

Several mechanisms have previously targeted the inhibition of Aβ production, namely through inhibition of β-site amyloid protein cleaving enzymes 1 and 2 (BACE1 and BACE2), and γ-secretase. BACE1, which is an enzyme from the aspartyl protease class, is the major β-secretase in the brain [30].

In transgenic mouse models of AD, BACE1 inhibitors dose-dependently lowered Aβ levels in the brain and CSF; however, very few studies have shown the ability to reduce the memory and behavioral deficits in such mouse models [30, 31]. Many BACE1 inhibitors clinical trials showed significantly lowered Aβ levels in the plasma and CSF, and reduced brain amyloid loads but without cognitive, clinical, or functional benefit [30]. A few of these clinical trials were terminated prematurely due to toxicity or cognitive and behavioral worsening compared to placebo-treated patients [30]. Elenbecestat was the last BACE1 inhibitor in phase III clinical trials (NCT02956486) [30]. However, the sponsors, Eisai and Biogen, reported during a press release the discontinuation of the phase III clinical trial due to “unfavorable risk-benefit ratio” [30]. Other BACE1 inhibitors such as LY3202626 (Eli Lilly & Co) and umibecestat/CNP520 (Novartis Pharmaceuticals Corporation and Amgen, Inc.) were also abandoned due to no clear effects on cognition for LY3202626 and worsened cognition, brain atrophy and weight loss for umibecestat [30]. There are currently no BACE1 inhibitors in active clinical trials [30].

Drugs targeting γ-secretase also showed unfavorable results in clinical trials. For example, a phase II clinical trial for avagacestat (Bristol-Myers Squibb) given orally showed no evidence of efficacy and was associated with adverse dose-limiting effects (predominantly gastrointestinal and dermatologic) [32, 33]. Many γ-secretase inhibitors were also associated with significantly increased risk of serious adverse events (SAEs) such as skin cancers and cognitive decline. The high frequency of AEs associated with these drugs is making them less likely to be widespread agents for the pharmacological treatment of AD [34].

Since γ-secretase inhibition was unsuccessful, some efforts were getting directed towards γ-secretase modulators instead, which were expected to be safer as they target the γ-secretase complex through allosteric binding and modify its enzymatic activity instead of competing for substrates [35]. In pre-clinical settings, γ-secretase modulators such as rofecoxib (Merck), tarenflurbil (Myriad Genetics), or naproxen (Procter & Gamble) were able to reduce levels of Aβ_42_ and produce shorter peptides that are non-amyloidogenic [36–38]. However, γ-secretase modulators failed to show efficacy in clinical trials, which was explained by the very poor blood-brain barrier (BBB) crossing ability of these drugs [38]. Nonetheless, a new generation of γ-secretase modulators is currently under pre-clinical investigation [33479693].

### Aβ Agglomeration Inhibitors

Agents such as metal chelators, resveratrol, cromolyn sodium, and ibuprofen have been explored as potential agglomeration inhibitors. However, and as detailed below, as the date of writing of this paper, none of these agents have shown any substantial effects on improving cognitive abilities in AD clinical trials.

Deferiprone (Chiesi Pharmaceuticals Inc. and Apotex Inc.) is an iron chelator that is undergoing a phase II clinical trial (NCT03234686). The reasoning behind iron chelation is that Fe^3+^ is able to bind to Aβ_42_ via His 6, Asp7, Tyr10, and His14, which all facilitate Aβ aggregation [39 40]. Previously, the metal chelator clioquinol (also called iodochlorhydroxyquin or PBT1; Prana Biotechnology Limited) was being developed. But it was terminated due to a toxic contaminant [39]. The second-generation compound following PBT1, termed PBT2, had improved BBB crossing abilities and pharmacokinetics compared to PBT1. PBT2 reduces extracellular copper and zinc ions by translocating them into the cells and thus reduces metal-mediated Aβ aggregation [40–42]. PBT2 underwent two phase II clinical trials. The first one (NCT00471211) showed a decrease in Aβ_42_ in CSF but no cognitive improvement. The second one (ACTRN12611001008910) showed no difference between the treatment and placebo groups [43].

Resveratrol is a polyphenol found in some more than 70 plant species, most predominantly in grapes’ skin and seeds, and even in red wine [44]. The reasoning behind using resveratrol for AD treatment is that it can inhibit oxidative stress and activate the anti-aging gene *SIRT1*, which both are associated with decreased Aβ deposition [45, 46]. For example, Tg19959 mice treated with resveratrol showed a significant reduction in Aβ plaque formation by up to 90% [45]. There is currently one active phase I clinical trial for resveratrol that is recruiting patients with mild cognitive impairment, and patients that are pre-diabetic or type II diabetes mellitus (NCT02502253). The reasoning why pre-diabetic and diabetic patients are investigated is because high glucose levels increase the risk of incident cognitive impairment and possibly AD [47].

ALZT-OP1 (AZTherapies, Inc.) is a combination of cromolyn sodium and ibuprofen that is used in clinical trials to assess effects on Aβ plaque aggregation [48]. There is currently one active phase I/II clinical trial (NCT04570644) for ALZT-OP1 assessing the effects on AD patients and healthy volunteers. Cromolyn sodium is a prescription drug used in the treatment of asthma, and its mechanism of action is inhibition of mast cell degranulation, hence the modulation of inflammatory events [48]. Ibuprofen is a non-steroidal anti-inflammatory drug (NSAID) that inhibits cyclooxygenase (COX) 1 and 2. Although ibuprofen alone had no significant cognitive effect, cromolyn alone or combined with ibuprofen was found to inhibit the deposition of Aβ via the promotion of microglia recruitment and phagocytosis [48, 49].

### Immunotherapies as Aβ Deposition Inhibitors and Plaques Dissolution Agents

Since it was reported in 1999 that active immunization against Aβ reversed amyloid pathology in transgenic mice, academia and industry have worked intensively on the development of passive and active anti-Aβ immunotherapeutics for AD [50].

Active immunization entails a vaccination approach with the administration of Aβ as the antigen to elicit an immune response against Aβ [51]. Such an approach was first pursued in 2002 by administering pre-aggregated Aβ_42_ along with the immunological adjuvant QS-21 [51]. Although this innovative paradigm significantly reduced Aβ brain deposits in AD patients, it did not produce any cognitive or clinical benefits [51].

Monoclonal anti-Aβ antibodies (mAbs) are passive immunotherapy initiatives that have been investigated thoroughly as a treatment for AD. The current armamentarium of mAbs differs in selectivity for polymorphic variants and may recognize epitopes either based on a specific portion of the Aβ sequence, or one of the multimeric Aβ conformations [52]. Although up until early 2019 all mAb therapeutics failed in phase III clinical trials, the relative “success” of aducanumab with their phase III study that was reported in December of 2019 rendered some excitement among the AD researchers and patients [53]. Furthermore, the recent reports on donanemab are reinvigorating hope for the use of anti-Aβ mAbs in the treatment of AD. Below, we describe the major immunotherapies applied to lower brain Aβ levels that are still currently investigated in clinical trials. The current mABs are summarized in Table 1.

**Table 1:** Properties of selected anti-Aβ antibodies currently tested in clinical trials for AD

mAb clinical candidate	Mouse antibody analog	Clinical stage and status	Aβ selectivity (monomer, aggregate)	Epitope (residues)	Sponsor
Aducanumab	aducanumab	Phase III, Enrolling by invitation	A>>M	3-7	Biogen Inc.
Lecanemab (BAN2401)	mAb158	Phase III, recruiting	A>>M	1-16	Biogen Inc. and Eisai Co.
Solanezumab	M266	Phase III, recruiting	M>>A	16-26	Eli Lilly and Co.
Crenezumab (MABT5102A)	MABT5102A	Phase III, terminated	A=M	13-24	Genentech Inc.
Donanemab	mE8-IgG2a	Phase II, recruiting	A>M	N-terminal pyroglutamate	Eli Lilly and Co.
Gantenerumab	gantenerumab	Phase III, recruiting	A>M	3–11,18–27	Hoffman-La Roche Inc.

### Aducanumab

Aducanumab (ADU) is currently one of the most promising mAbs approaching approval by the U.S. Food and Drug Administration (FDA). ADU is a recombinant human IgG1 antibody that primarily binds to both soluble and insoluble Aβ amyloid aggregates with > 10,000-fold selectivity over monomers [52]. It was derived from a blood lymphocyte library collected from a healthy donor population of elderly subjects who were lacking signs of cognitive impairment or with unusually slow cognitive decline [52]. ADU binds to Aβ residues 3–7 in an extended conformation [52]. It is capable of selectively targeting the pathological oligomeric and fibrillar forms of Aβ [52].

Preclinical studies in Tg2576 mice have shown reduced Aβ plaque size in a dose-dependent manner in young (9 months old) but not aged (22 months old) animals [54, 55], suggesting this mAb prevents Aβ aggregation more than it helps sorbing existing plaques. To note, however, this reduction in Aβ plaques was not accompanied by any cognitive or behavioral improvement [55].

A phase Ia clinical trial (NCT01397539) completed in 2016 tested single ascending intravenous doses of aducanumab in 53 AD patients to evaluate the safety, tolerability, and pharmacokinetics [56]. Low doses of ≤30 mg/kg did not show SAEs [56]. All three patients receiving a 60 mg/kg ADU developed SAEs consisting of amyloid-related imaging abnormalities (ARIA) [56]. None of the patients discontinued or withdrew from treatment due to SAEs, and all SAEs completely resolved within 8–15 weeks following the single 60 mg/kg dose administration, which was the final titration dose [56]. Interestingly, at the 60 mg/kg dose, Aβ_40_ and Aβ_42_ levels increased in the plasma for ~3 weeks, suggesting that high levels of ADU bind to soluble monomeric Aβ in humans [56]. However, after 24 weeks of treatment, there was no significant difference in cognitive abilities compared with placebo as measured by the 13-item Alzheimer’s Disease Assessment Scale–Cognitive (ADAS-Cog13) [56], positively demonstrating the absence of toxicity on cognition due to ADU.

Analysis of the phase Ib study, PRIME (NCT01677572) showed a significant reduction in brain Aβ loads in prodromal or mild AD subjects when monitored via florbetapir positron emission tomography (PET) imaging. The results were dose- and time-dependent when observed over one year of monthly intravenous infusions [54]. The PRIME analysis also showed that ADU injections result in a slowing of clinical decline at 1 year, as measured by Mini-Mental State Examination (MMSE) and Clinical Dementia Rating Scale-Sum of Boxes (CDR-SOB) [54].

ADU recently underwent two large phase III clinical trials dubbed “ENGAGE” (NCT02477800) and “EMERGE” (NCT02484547). Both studies were conducted on individuals showing signs of mild cognitive impairment and mild dementia due to AD. The trials used the CDR-SOB as their primary endpoint measurement [57•]. Unfortunately, both trials were terminated in March 2019 by the sponsor, Biogen, due to interim post hoc analyses showing “futility.” The data showed EMERGE trending positive and ENGAGE unlikely to meet its primary endpoints. Later in October 2019, Biogen held a press conference to announce that further analyses suggest the benefits of high-dose (100 mg/kg) ADU in both trials. This prompted Biogen to formally submit a new request for drug approval to the FDA [57•]. However, Biogen claims have been received with some skepticism. For example, Knopman et al. recommended running another trial using high-dose ADU of at least 78 weeks in duration [58]. This recommendation is suggested since post hoc analyses can be fickle and unreliable, indicating that more information needs to be collected to strengthen the existing data before seeking FDA approval [58].

ADU is currently in the pipeline to be reviewed by the FDA [59]. However, a medical advisory committee convened by the FDA did not recommend approval yet, based upon skepticism of adequacy of existing evidence of efficacy [59]. Currently, Biogen has another phase III trial (NCT04241068), which is recruiting patients who were participating in one of the previous ADU studies at the time of the announcement of early termination of ENGAGE and EMERGE.

### Lecanemab

Lecanemab (BAN2401) is a humanized IgG1 version of the mouse monoclonal antibody mAb158. It selectively binds to large, soluble Aβ protofibrils [60]. Preclinical studies have demonstrated its ability to decrease levels of pathogenic Aβ, prevent Aβ deposition, and selectively reduce Aβ protofibrils in the brain and CSF in AD animal models [61, 62].

Based on favorable preclinical findings, as well as phase I (NCT02094729) and II (NCT01230853) clinical trial results [60], multiple trials are currently investigating BAN2401 as a potential viable treatment option for AD. For example, ClarityAD (NCT03887455) is a phase III randomized, placebo-controlled, double-blind, parallel-group trial that is actively recruiting participants with mild cognitive impairment due to AD. The aim of the study is to evaluate the efficacy of lecanemab in participants with early Alzheimer’s disease (EAD) by determining the superiority of lecanemab compared with placebo on the change in cognition from baseline via the CDR-SOB. In this trial, lecanemab 10 mg/kg will be administered intravenously once every 2 weeks. The anticipated completion date for ClarityAD is June 2022.

Another actively recruiting trial investigating BAN2401 is the AHEAD3-45 trial (NCT04468659). This study aims to the evaluate efficacy and safety of lecanemab in patients with preclinical AD, such as having a first-degree relative diagnosed with dementia onset before age 75, possessing at least one apolipoprotein E4 (*APOE4*) allele, or elevated amyloid levels in the central nervous system (CNS) demonstrated by previous amyloid PET imaging or CSF measurements. Participants will receive lecanemab 5 mg/kg, administered as intravenous (IV) infusions every 2 weeks through 8 weeks, then 10 mg/kg administered as IV infusions every 2 weeks through 96 weeks, and 10 mg/kg administered as IV infusions every 4 weeks through 216 weeks.

Results from these trials will offer clinical evidence to determine whether lecanemab is a robust anti-Aβ agent in humans as was observed in murine-based studies. Additional long-term trials such as ClarityAD and AHEAD3-45 are needed to continue the quest for definitive clinical outcome results in individuals with early AD.

### Solanezumab

Solanezumab is a humanized monoclonal antibody that preferentially binds to the mid-region of the Aβ peptide and reduces brain Aβ burden by altering CNS and plasma Aβ clearance in transgenic mouse models of AD [63, 64]. This is achieved by solanezumab sequestering all plasma Aβ and creating an efflux of CNS Aβ into the plasma, thus causing a decrease in CSF Aβ levels [65]. Two previous clinical trials investigating solanezumab have been completed, i.e. Expedition 1 (NCT00905372) and Expedition 2 (NCT00904683), while another two trials were recently terminated, i.e., Expedition 3 (NCT01900665) and ExpeditionPRO (NCT02760602). The primary objective of each study was to slow down cognitive decline in patients with mild dementia due to AD [66]. More specifically, Expedition 3 was terminated due to the failure of solazenumab to significantly reduce cognitive decline in patients with mild AD dementia [67]. Further, ExpeditionPRO was terminated due to insufficient scientific evidence that solanezumab would likely demonstrate a meaningful benefit to participants with prodromal AD.

Resulting from the termination of the Expedition studies, researchers have continued to search for a definitive answer on the efficacy of solanezumab. The only trial currently enrolling participants is the DIAN-TU trial, a phase II/III randomized, double-blind, placebo-controlled study which aims to assess whether IV infusion of solanezumab slows the rate of progression of cognitive impairment and improves disease-related biomarkers in individuals with mutations causing dominantly inherited AD (NCT01760005). In this parallel assignment study, solanezumab administered every 4 weeks at escalating doses will be compared with gantenerumab, which is a fully humanized IgG1 that has demonstrated high-affinity binding to cerebral Aβ and to significantly reduce Aβ plaques in both AD transgenic mouse models [68••] and after 2 years of treatment in humans [69•]. Efficacy will be measured by the change from baseline in the DIAN-multivariate cognitive endpoint which consists of four measures: Wechsler Memory Scale-Revised Logical Memory Delayed Recall Test, Wechsler Adult Intelligence Sale Digit Symbol Substitution Test (WAIS), International Shopping List Task (ISLT), and MMSE. This DIAN-TU study is expected to enroll 490 participants and is projected to reach completion in July 2022. Building on findings from previous clinical studies, this trial, and future trials will assist in determining tolerability, toxicity, and adequate dosing for solanezumab in the AD population. Additionally, the efficacy of solanezumab will be determined as more studies with improved designs investigate its Aβ-lowering abilities.

### Crenezumab

Crenezumab (MABT5102A) is a humanized anti-Aβ monoclonal IgG4 with affinity to multiple Aβ species, especially for pentameric and fibrillary 16-mer assemblies of aggregated Aβ [70, 71]. Consequently, crenezumab is able to bind both monomeric and aggregated forms of Aβ. Crenezumab also possesses anti-aggregative properties towards Aβ, promotes disaggregation, and protects neurons from oligomer-induced cytotoxicity [70, 72]. Crenezumab was created based on the hypothesis that the human IgG4 constant region would modify Fc effector function and reduce vascular side effects [70].

Preclinical studies in Tg2576 mice showed no inflammatory response following intracerebral injection [72]. A completed phase Ib study (NCT02353598) called GN29632 demonstrated tolerability of ≤ 120 mg/kg doses administered intravenously every 4 weeks [73]. Although ~94% of participants in GN29632 experienced at least one adverse event (AE), most AEs were mild or moderate [73]. Only 4.9% (double-blind treatment period) and 9.9% (combined double-blind treatment and open-label extension periods) showed new ARIA-micro hemorrhages and hemosiderosis (ARIA-H), which was not enough to identify any new prominent safety issues [73].

Completed phase II clinical trials of crenezumab in patients with mild-to-moderate AD include ABBY (NCT01343966) and BLAZE (NCT01397578) studies [74, 75]. The primary objective for ABBY was an improvement in ADAS-Cog12 and CDR-SOB scores from baseline to week 73 [74]. The primary objective for BLAZE was a favorable change in Aβ burden from baseline to week 69 as measured by florbetapir PET in the modified intent to treat population [75]. Additionally, secondary outcomes for BLAZE were changes in CSF biomarkers and fluorodeoxyglucose PET from baseline to week 69, and changes in ADAS-Cog12 and CDR-SOB from baseline to week 73 [75]. Although neither of these two studies met their primary or secondary endpoints, positive post hoc analyses in a subset of patients with very mild AD and treated with high dose validates the idea of testing high-dose crenezumab in patients in an early stage of AD [74]. Testing with a higher dose is also supported by a phase Ib study (GN29632) [76].

Two recent phase III studies, i.e., CREAD (NCT02670083) and CREAD2 (NCT03114657), have investigated the efficacy and safety of crenezumab at 60 mg/kg, which is 4 times higher than the previous phase II trials. These studies were discontinued following the interim analysis of CREAD, which showed unlikeliness to meet the primary endpoint of change from baseline to week 105 in the CDR-SOB score [76, 77]. The overall safety profile was similar to that seen in previous trials with no obvious safety signals [76].

There is currently one active phase II clinical trial for crenezumab that is recruiting patients in the preclinical phase of AD that carry the presenilin 1 (PSEN1) E280A autosomal dominant mutation (NCT01998841). This study is scheduled for completion in February 2022.

### Donanemab

The latest and most promising mAb against Aβ is donanemab (LY3002813, or N3pG). It is a humanized IgG1 that reduces amyloid plaques in AD by targeting Aβ(p3-42), which is an N-terminal pyroglutamate Aβ epitope [78]. Lowe et al. recently investigated donanemab in patients with MCI due to AD. Donanemab demonstrated general safety and tolerability in this double-blind, randomized, placebo-controlled, parallel-group, single-dose followed by a multiple-dose, dose-escalation study [79••]. Patients were assigned to five dosing cohorts, ranging from 0.1 to 10 mg/kg, or a placebo cohort followed by a 12-week follow-up period for each dose. Interestingly, amyloid PET showed that the 10-mg/kg dose led to brain amyloid load reduction of 40–50% [79••].

Recent results from the TRAILBLAZER-ALZ trial highlighted donanemab as a promising mAb treatment of early symptomatic AD [80]. In this multicenter, randomized, double-blinded, placebo-controlled phase II trial, Mintun et al. assessed whether donanemab administration would improve cognition in patients with prodromal or mild AD. For up to 72 weeks, patients were intravenously administered either 700 mg donanemab (~10 mg/kg) or placebo for the first three doses and 1400 mg (~20 mg/kg) thereafter every 4 weeks. The primary outcome of TRAILBLAZER-ALZ was the change from baseline on the iADRS at 76 weeks. Secondary outcomes included change in scores on the CDR-SB, ADAS-Cog13, MMSE, and Alzheimer’s Disease Cooperative Study - Instrumental Activities of Daily Living (ADCS-iADL), along with changes in amyloid and tau levels. At 76 weeks, the donanemab group demonstrated significant improvement on the Integrated Alzheimer’s Disease Rating Scale (iADRS) over the placebo group. While donanemab did not lead to improvement in most secondary outcomes, florbetapir and tau PET scans illustrated significant decreases in brain amyloid and tau loads, respectively. These results show the ability of donanemab to positively affect the cognitive and functional decline in early symptomatic AD, which could be applied to other stages of AD.

As a follow-up to the TRAILBLAZER-ALZ study, Eli Lilly and company are currently recruiting participants for a phase II, randomized, parallel assignment study investigating intravenous donanemab in early symptomatic AD (NCT04437511). The primary outcome measure of the TRAILBLAZER-ALZ2 study is the change from baseline on the CDR-SOB. An important inclusion criterion is a gradual and progressive change in memory function reported by participants or informants for ≥ 6 months. The trial is estimated to reach completion in 2024. It is our opinion that research efforts should place additional focus on donanemab, since it is showing the highest potential as an Aβ-lowering agent accompanied by cognitive improvement among all immunotherapies investigated to date. Future trials will ultimately determine the efficacy of this novel mAb in the AD population.

### Gantenerumab

Gantenerumab is a human IgG1 antibody that binds to aggregated Aβ in the brain and lowers amyloid-β by eliciting effector cell-mediated clearance [81]. Klein et al. recently reported that gantenerumab doses up to 1200 mg administered subcutaneously once every 4 weeks demonstrated significant Aβ removal in patients with prodromal to moderate AD [69•]. The main endpoint of this open-label study was the change in the Aβ plaque burden from baseline to week 52 and week 104. Florbetapir PET was used to assess the efficacy of gantenerumab in Aβ plaque reduction. Earlier this year, Klein et al. reported that subcutaneous gantenerumab doses up to 1200 mg continued to reduce Aβ plaque burden at 36 months following treatment initiation [68••].

In addition to the DIAN-TU phase II trial (NCT01760005), gantenerumab is being investigated in a phase II, multicenter, open-label, single-arm, pharmacodynamic study in participants with early AD (NCT04592341). The study is currently recruiting and is planned to be completed in February 2024. Its primary outcome measure is the change from baseline to week 104 in brain amyloid as measured by brain amyloid PET centiloid levels. Enrolled patients will initially be administered a single subcutaneous injection of gantenerumab 120 mg once every 4 weeks for 12 weeks, followed by 255 mg every 4 weeks for 12 weeks, and 255 mg every 2 weeks for another 12 weeks, followed by 255 mg once every week for up to week 103.

A randomized, double-blind, placebo-controlled, parallel-group phase III study is currently recruiting participants for a study investigating gantenerumab in early AD (NCT03444870). The study will evaluate the efficacy and safety of gantenerumab versus placebo in participants with early AD and is planned to be completed in November 2023. The primary outcome measure is the change from baseline to week 116 in CDR-SOB score. Trial eligibility includes a diagnosis of probable AD dementia or prodromal AD, evidence of the AD pathological process as confirmed by CSF tau/ Aβ_42_ or amyloid PET scan, and demonstrated abnormal memory function. Findings from this study combined with results from previous studies will determine future investigations of gantenerumab in the prevention and treatment of AD.

## Conclusion

Highly specific mAbs targeting Aβ are positioned to lead a new generation of disease-modifying therapies for AD. We have assessed the current mAb drug development landscape, which continues to offer additional therapeutic options. If the FDA approves aducanumab, this would mark a turning point in the drug development landscape since no drug has been approved for the treatment of AD since 2003. Data from aducanumab studies of EMERGE and ENGAGE makes consideration of approval feasible. First, aducanumab resulted in a significant 22% slowing of decline on the CDR-sb^.^ Second, other measures including the Mini-Mental State Examination, Alzheimer’s Disease Assessment Scale -cognitive subscale, and mild cognitive impairment (MCI) version of the Alzheimer’s Disease Cooperative Study Activities of Daily Living scale (ADCS ADL) demonstrated statistically significant drug-placebo differences in favor of active therapy. Third, care partners reported 84% less associated distress at week 78 compared to care partners of those on placebo. Finally, in ENGAGE AND EMERGE, participants who received the highest dose of aducanumab for 14 months showed similar levels of slowing on the CDR-sb (30% slowing in EMERGE, 27% slowing in ENGAGE).

While approval would offer a much-anticipated treatment option to millions of patients, rejection by the FDA would leave a void in the toolkit of physicians who desperately desire additional options to offer their patients, and a tremendous level of uncertainty in AD patients and their caregivers. Limited positive outcomes in trials due to the small number of studies investigating other mAbs in AD such as lecanemab, solanezumab, crenezumab, donanemab, and ganterenumab should be further investigated in additional studies.

Each of the mAbs we discussed here has proven to be relatively safe in humans. Results from the phase III trials of ClarityAD, CREAD, and CREAD2 will offer details on the efficacy of their respective drug in improving cognitive and functional impairment as well as imaging indicators of amyloid presence, thus contributing to the growing evidence surrounding mAb viability in treating neurodegeneration. Future directions should focus on bidirectional studies that may ascertain mechanisms by which immunotherapy leads to improvements in AD.

## Data Availability

Not applicable
